# Spatial patterns of clinically significant G6PD deficiency: variants, prevalence and an overall national risk index

**DOI:** 10.1186/1475-2875-11-S1-P49

**Published:** 2012-10-15

**Authors:** Rosalind E Howes, Simon I Hay

**Affiliations:** 1 Spatial Ecology and Epidemiology Group, Department of Zoology, University of Oxford, OX1 3PS, UK

## Background

Threat of haemolytic anaemia in individuals with a deficiency in glucose-6-phosphate dehydrogenase (G6PD) enzyme activity prevents widespread use of primaquine, a vital drug for malaria elimination. Significant genetic variation underpins this human enzyme disorder, reflected as a spectrum of clinical severity following exposure to oxidative agents such as primaquine. Across malaria endemic countries (MECs), the prevalence and genetic basis of this predisposing condition vary greatly. An understanding of the relative risks presented by G6PD deficiency, in terms of prevalence and severity, is necessary to rationally determine primaquine drug policy.

## Materials and methods

We assembled a geo-referenced database documenting the spatial distribution of G6PD variants. A suite of maps were developed to illustrate this current state of understanding, including occurrence maps of the commonly reported variants and, where population samples were locally representative, maps detailing the relative prevalence of co-occurring variants. Countries were stratified according to the relative severity of the variants reported. These categorisations were combined with ranks of the overall frequency of deficiency in each country, resulting in an overall index of G6PD deficiency associated risk. A parallel index of uncertainty in the index was also developed.

## Results

The variant maps presented here make apparent the important contrasts between the spatial variants of G6PD deficiency between populations. As well as diversity among the variants identified within populations, there is great heterogeneity between the dominant variants identified between different populations. The Mediterranean variant was found to be the predominant variant in MECs across west Asia, where prevalence of deficiency was also high; these countries therefore ranked highest on the risk index. Across the Asian continent, risk from G6PD remained high due to the severity of reported variants and the disorder being relatively common. The low severity of variants reported from sub-Saharan African countries resulted in lower risk categorisation relative to Asia, though uncertainty in the data from this continent was high.

## Conclusions

Developing an evidence-base to support primaquine drug policy is essential. We present a cartographic suite and a framework for stratifying risk for regional comparisons. The risk index proposed here required several assumptions to overcome significant knowledge gaps. A deeper understanding of the common variants’ primaquine sensitivity phenotypes is essential to maximise safe and effective use of primaquine.

